# Differentiation between 3,4- and 4,15-Epoxyeudesmanolides by Electrospray Ionization Tandem Mass Spectrometry

**DOI:** 10.1155/2017/7921867

**Published:** 2017-11-06

**Authors:** Herbert Júnior Dias, Ricardo Stefani, José Carlos Tomaz, Ricardo Vessecchi, Antônio Eduardo Miller Crotti

**Affiliations:** ^1^Departamento de Química, Faculdade de Filosofia, Ciências e Letras de Ribeirão Preto, Universidade de São Paulo, Ribeirão Preto, SP, Brazil; ^2^Instituto de Ciências Exatas e da Terra, Universidade Federal do Mato Grosso, Campus Universitário do Araguaia, Araguaia, MT, Brazil; ^3^Departamento de Física e Química, Faculdade de Ciências Farmacêuticas de Ribeirão Preto, Universidade de São Paulo, Ribeirão Preto, SP, Brazil

## Abstract

We investigated the fragmentation of the eudesmanolide-type sesquiterpene lactones 1*α*-(4-hydroxymethacryloyloxy)-3*α*,4*α*-epoxy-8*α*-hydroxyeudesm-11(13)-6*α*,12-olide (**1**) and 1*α*-(2,3-epoxyangeloyloxy)-4*α*,15-epoxy-8*α*-hydroxyeudesm-11(13)-6*α*,12-olide (**2**) by electrospray ionization tandem mass spectrometry (ESI-MS/MS). The elimination of the different ester substituent at C(1) directly from protonated** 1** and** 2** (**A**) led to the formation of two regioisomer product ions** B** (**A** − RCO_2_H). Further fragmentation of** B** resulted from consecutive eliminations of H_2_O and CO molecules. However, we identified four product ions that allowed for the differentiation between 3,4- and 4,15-epoxyeudesmanolides. The formation of these diagnostic ions was associated with the C(3)–O bond of compound** 1**, which propitiates the participation of the lone pair of the oxygen epoxide in the formation of** B** through a Grob-Wharton-type fragmentation, then resulting in an alternative fragmentation pathway. These data can be useful for the fast differentiation between epoxyeudesmanolide regioisomers directly from* Dimerostemma *extracts by liquid chromatography-tandem mass spectrometry (LC-MS/MS), as an alternative to NMR, or even for quantitation studies of these compounds using multiple reaction monitoring (MRM) scan.

## 1. Introduction

Eudesmanolides are sesquiterpene lactones (STL) biosynthetically derived from farnesyl pyrophosphate, whose occurrence has been widely reported in plants of the Asteraceae family [1]. These compounds display a wide spectrum of biological activities, such as antitumor [2], antiproliferative [3], anti-inflammatory [4], antifungal [5, 6], antimicrobial [7], insecticidal [8], and inhibitory activity of the protein PTP1B, responsible for type 2 diabetes and obesity [9].

Structurally, eudesmanolides exhibit a decalin system fused to a *γ*-lactone ring. Although* trans-*fused eudesmanolides are the most common forms,* cis*-fused eudesmanolides may also occur [1]. The structural diversity of these compounds emerges mainly from oxidations in the basic sesquiterpene skeleton [1, 10]. The enzymatic oxidation of the double bonds of eudesmanolides along the biosynthetic process results in the formation of epoxyeudesmanolide regioisomers, whose structural elucidation often requires a detailed (and sometimes exhaustive) analysis of the ^1^H and ^13^C nuclear magnetic resonance (NMR) spectra [11].

Over the last two decades, electrospray ionization tandem mass spectrometry (ESI-MS/MS) has proved to be a powerful analytical technique for the fast identification of natural products [12, 13]. In some specific cases, the high sensitivity and selectivity, as well as the possibility of using nondeuterated solvents, have rendered ESI-MS/MS even more attractive than NMR [14]. Moreover, the coupling of liquid chromatography to electrospray ionization tandem mass spectrometry (LC-ESI-MS/MS) has allowed for the quantification of these compounds directly from crude extracts and complex matrices [14, 15]. It is noteworthy that the sensitivity and the selectivity of LC-MS/MS can be increased significantly by using the multiple reaction monitoring (MRM) scan mode, which is based on two or more transitions from the precursor ion to specific (and eventually diagnostic) product ions [16]. However, the fragmentation patterns of several classes of natural products under collision-induced dissociation (CID) conditions have not been well-established yet, which has made building spectral libraries difficult and limited the use of LC-ESI-MS/MS for qualitative and quantitative studies on these compounds [12].

As part of our ongoing project on the gas-phase fragmentation reactions of natural products and their analytical applications [17], in this study, we will report the fragmentation of two 3,4- and 4,15-epoxyeudesmanolides using ESI-CID-MS/MS and its potential to distinguish these compounds.

## 2. Material and Methods

### 2.1. Mass Spectrometry Analysis

**Figure 1:**
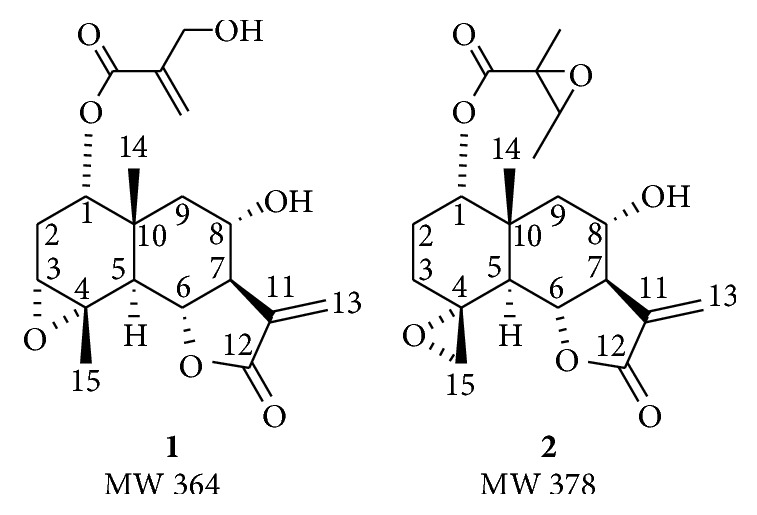
Structure of the eudesmanolides** 1** and** 2**.

The epoxyeudesmanolides** 1** (1*α*-(4-hydroxymethacryloyloxy)-3*α*,4*α*-epoxy-8*α*-hydroxyeudesm-11(13)-6*α*,12-olide) and** 2** (1*α*-(2,3-epoxyangeloyloxy)-4*α*,15-epoxy-8*α*-hydroxyeudesm-11(13)-6*α*,12-olide) (Figure 1) were previously isolated from* Dimerostemma vestitum* [11] and* Dimerostemma rotundifolium *[18].

Compounds** 1** and** 2** were dissolved in methanol/water 4 : 1 (v/v) and analyzed on a Quattro-LC instrument (Micromass, Manchester, UK), provided with an electrospray ion source operating in the positive ion mode and a triple quadrupole (QqQ) mass analyzer. Solutions of the compounds (0.3 mg·mL^−1^) were infused into the ESI source at a flow rate of 5 *μ*L·min^−1^ using a Harvard Apparatus model 1746 (Holliston, MA) syringe pump. The desolvation and ionization source block temperatures were at 250 and 120°C, respectively. The capillary and the cone voltage were optimized to 3.0 kV and 15 V, respectively. Collision-induced dissociation (CID) was performed on the isolated protonated molecule using argon collision gas (7 psi) and nitrogen was used as desolvation and nebulizing gas (flow rates of 260 and 26 L·h^−1^, resp.). The analytical mass range was 40–400 *m*/*z*. In labeled hydrogen experiments, deuterium oxide (D_2_O, 99.9 atom % D, Aldrich®) was added to the samples in a proportion of 10% v/v. The product ion spectra were obtained using collision energy ranging from 0 to 30 eV.* Quasi*-MS^3^ experiments were carried out by selecting the product ions generated by in-source dissociation and using them as precursor ions in CID experiments.

### 2.2. Computational Methods

The conformational analyses of the epoxyeudesmanolides** 1**-**2** were obtained by estimation of most stable conformers and optimization of geometries applying MM2 force field. The lowest energy conformers were reoptimized on the basis of DFT calculations at mPW1PW91/6-31+G(d) level, employing Gaussian 03 software [19]. The stationary point was estimated through vibration frequencies calculation at the same model. The protonation sites of the epoxyeudesmanolides** 1**-**2** were investigated employing the proton affinity (PA) as descriptor, as previously reported [20]. The enthalpy of the proton for the reaction M + H^+^ → MH^+^ was considered as 1.48 kcal·mol^−1^ at 298 K [21]. Molecular electrostatic potential maps (MEP) were also plotted employing Molekel software [22].

## 3. Results and Discussion

### 3.1. Structure-Fragmentation Relationships

**Figure 2:**
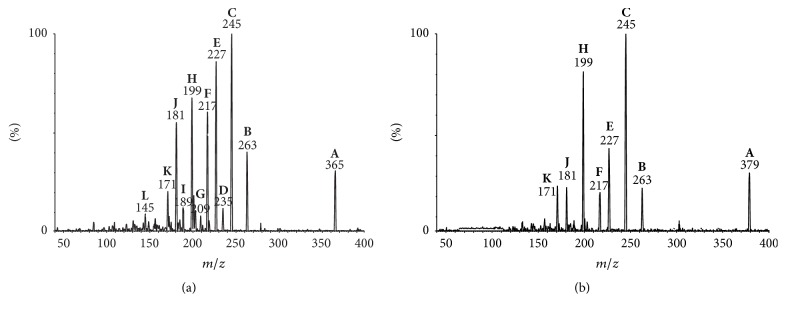
Product ion spectrum of the protonated** 1** (a) and** 2** (b) (*E*_lab_ = 15 eV).

Previous reports have demonstrated the importance of structure-fragmentation relationships for the rationalization of the gas-phase fragmentation pathways of compounds that exhibit the same structure core [12, 23–25]. However, to achieve trustable structure-fragmentation relationships, the product ion spectra must be obtained using the same collision energies (*E*_lab_). The optimum *E*_lab_ value must reduce the relative intensity of the precursor ion below 50% and maximize the intensity of other product ions without promoting extensive fragmentation [26]. In this work, the optimum *E*_lab_ for compounds** 1** and** 2** was found at 15 eV. The product ion spectra of protonated** 1** and** 2** at 15 eV are shown in Figure 2. The assignments of main product ions (relative intensity higher than 5%) of protonated** 1** and** 2** are given in Table 1.

**Scheme 1:**
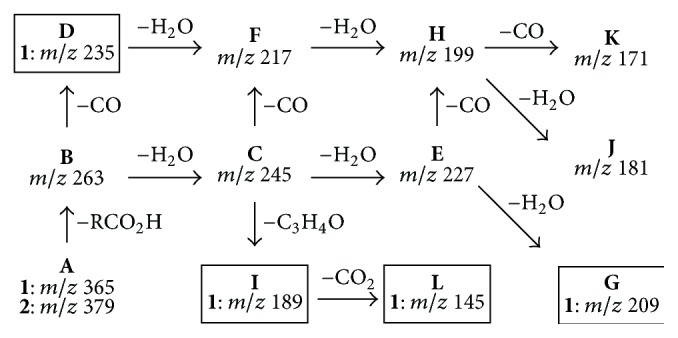
Structure-fragmentation relationships of protonated epoxyeudesmanolide-type sesquiterpene lactones** 1** and** 2**. The diagnostic product ions for compound** 1** (3,4-epoxyeudesmanolide) are shown within boxes.

The comparison between the product ion spectra of protonated** 1** (3,4-epoxyeudesmanolide) and** 2** (4,15-epoxyeudesmanolide), which differ in the acyloxy group at C(1), revealed a common product ion with *m*/*z* 263 (**B**). This ion results from the elimination of the corresponding carboxylic acids (4′-hydroxymetacrylic acid for** 1** and 2′,3′-epoxyangelic acid for** 2**) directly from the protonated molecule. Therefore, the product ion** B** of protonated** 1** and** 2** are regioisomers; consequently, differences between their fragmentations are expected to be due to the position of the epoxide ring in the basic structure of these eudesmanolides. A series of product ions derived from** B** by means of consecutive eliminations of H_2_O (18 u) and CO (28 u) are also common to** 1** and** 2**, such as** C** (**B** − H_2_O),** E **(**C** − H_2_O),** F** (**C** − CO),** H** (**E** − CO),** J** (**H** − H_2_O), and** K** (**H** − CO). This fragmentation pattern is similar to those previously reported for other sesquiterpene lactones [26]. On the other hand, the product ions** D** (*m*/*z* 235),** G** (*m*/*z* 209),** I** (*m*/*z* 189), and** L** (*m*/*z* 145) were observed only in the product ion spectrum of** 1**. Thus, these diagnostic ions can be useful to differentiate a 3,4-epoxyeudesmanolide (**1**) from a 4,15-epoxyeudesmanolide (**2**) isomer exhibiting the same structural core. Indeed, data from* quasi*-MS^3^ experiments supported that the product ions** F** (*m*/*z* 217) and** H** (*m*/*z* 199) can be formed by two different pathways. The ion** F** can be resulting from the elimination of CO from** C** (for** 1** and** 2**) or H_2_O from** D** (only for 1), whereas** H** can be formed by elimination of CO from** E** or H_2_O from** F** for both the compounds. An overview of the fragmentation pathways of protonated epoxyeudesmanolides** 1 **and** 2** allowed for identifying some diagnostic ions as depicted in Scheme 1.

### 3.2. Search for the Protonation Site

**Figure 3:**
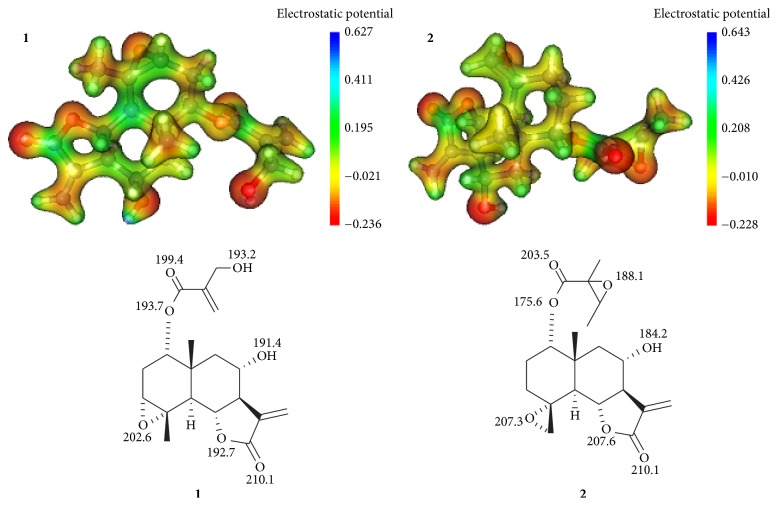
Molecular electrostatic potential maps (MEP) and proton affinity (PA) of epoxyeudesmanolides** 1**-**2**. All values of PA are in kcal·mol^−1^.

The determination of the protonation site has been reported as an important step for the rationalization of the gas-phase fragmentation pathways [25]. In principle, protonation can take place in many basic sites of the structure; however, an excess of the species containing the proton attached to the most basic site of the structure is expected. Upon CID conditions, two main possibilities must be considered: (a) the proton could remain bounded to the most basic site of compounds after the collision activation, so that the kinetic energy is only converted into internal energy [27] and (b) the proton is initially attached to the most basic site of molecule, but it can migrate to less basic sites upon CID process, then initiating the fragmentation process, in accordance with the “proton mobile model” [28]. In this study, we first used molecular electrostatic maps (MEPs, Figure 3) to identify the possible sites of the structures of the epoxyeudesmanolides** 1** and** 2**, in which the protonation can occur during the ionization process. The MEPs of these compounds (Figure 3) indicated the oxygen atoms as the most likely protonation sites, mainly the carbonyl oxygen [29, 30]. Next, the proton affinity (PA) values were estimated for all the protonation sites suggested by MEPs (Figure 3). The PA values indicated that the carbonyl oxygen of the *γ*-lactone ring of both epoxyeudesmanolides is the most susceptible sites to protonation.

In addition, the translational energy of the protonated epoxyeudesmanolides** 1** (*m*/*z* 365) and** 2** (*m*/*z* 379) converted into internal energy (*E*_com_ = *E*_lab_[*m*_*c*_/(*m*_*c*_ + *m*_*i*_)]) by the collision gas (argonium) at *E*_lab_ = 15 eV were calculated to 34.0 and 32.9 kcal·mol^−1^, respectively [31]. These data revealed that the energy provided upon CID is higher than the difference between the energies of all the protonation sites, as estimated by computational methods.

### 3.3. Formation of the General Ions B, C, E, F, H, J, and K

**Scheme 2:**
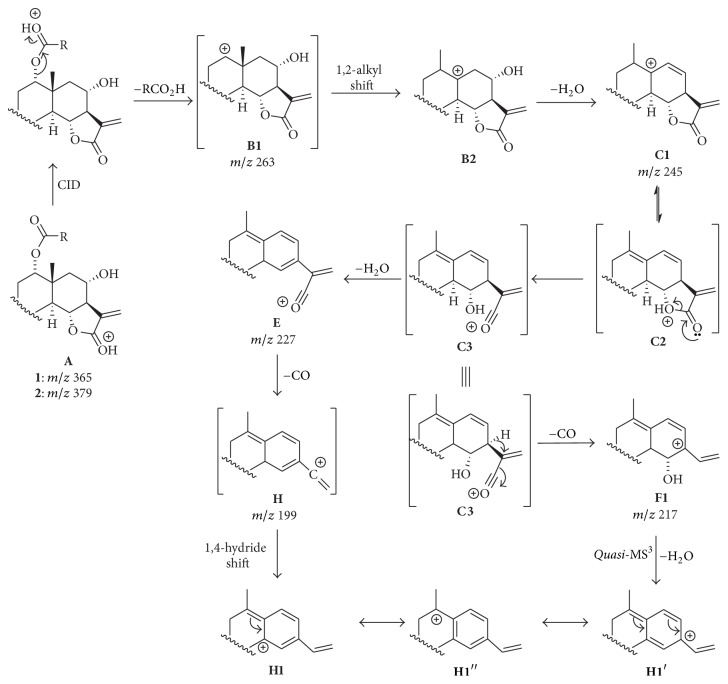
Formation of the general product ions** B**,** C**,** E**,** F,** and** H** of protonated** 1** and** 2**.

The product ion** B** (*m*/*z* 263) is formed from protonated** 1** and** 2** by means of the elimination of the ester side chain at C(1) as its corresponding carboxylic acid, as previously mentioned in this paper. Data from the hydrogen labeled experiments (Table 1) revealed that the deuterium/hydrogen attached to the molecule during the ionization process is involved in this step. These data evidenced that the elimination of the carboxylic acid must be preceded by the proton migration from the *γ*-lactone oxygen (the most basic site of the structure of** 1** and** 2**) to the carbonyl oxygen of the ester function at C(1). The energy difference needed to promote this migration is estimated to be 10.7 kcal·mol^−1^ for** 1** and 6.6 kcal·mol^−1^ for** 2**, as indicated by the PA values, which is lower than the *E*_com_ corresponding to *E*_lab_ = 15 eV. The elimination of side-chain group at C(1) by inductive cleavage results in the product ion** B1** (*m*/*z* 26), as shown in Scheme 2 [32]. Considering that** B1** is a secondary carbocation, its rearrangement to** B2** (a tertiary carbocation) by means of a 1,2-alkyl shift is driven by the charge stabilization [33]. Alkyl migrations driven by increase in the stability due to hyperconjugative effects have been also reported for similar compounds, such as steroids [34]. Elimination of H_2_O from** B2** by means of a remote hydrogen rearrangement leads to the formation of** C1** (*m*/*z* 245), which is the most intense product ion in the CID spectrum of the epoxyeudesmanolides** 1** and** 2 **[35].

Further fragmentation of** C1** is preceded by its conversion into** C2**, from which the opening of the *γ*-lactone ring occurs to produce the acylium ion** C3**, as previously reported for other *γ*-lactones (Scheme 2) [36]. The water elimination from** C3** produces** E** (*m*/*z* 227), which has an extended conjugation as compared to** C3**. Elimination of CO elimination from** E** results in the vinylic carbocation** H** (*m*/*z* 199), which can easily rearrange to the aromatic cation** H1** by means of a hydride shift from C(5) to C(11) [37]. On the other hand, the CO elimination from** C3** can be assisted by a 1,2-hydride shift from C(7) to C(11) to result in the product ion** F** (*m*/*z* 217). Finally, dehydration of** F1** also leads to** H1**, as confirmed by data from* quasi*-MS^3^ experiments (Scheme 2). The differences between the relative intensities of** F** in the product ion spectrum of** 1** (62%) and** 2** (19%) will be further discussed in this paper.

**Scheme 3:**
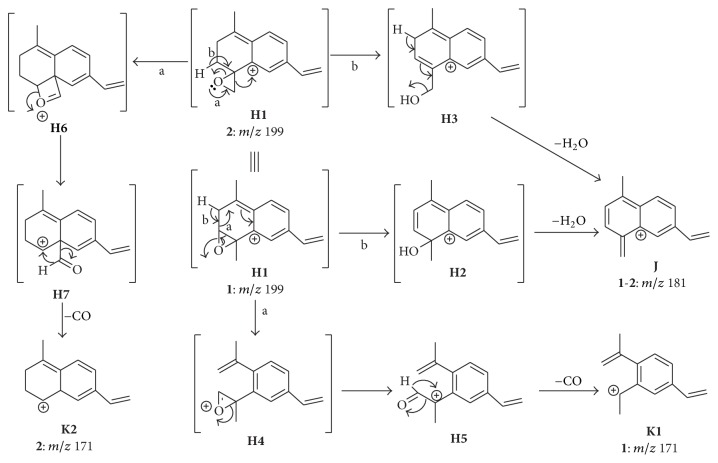
Formation of the general fragment ions** J **and** K **for compounds** 1** and** 2.**

Formation of the product ion** J** (*m*/*z* 181) for both** 1** and** 2** is preceded the cleavage of the C(3)–O of** H1** and consequent opening of the epoxide ring by means of remote hydrogen rearrangements (Scheme 3). On the other hand, although the product ions** K** (*m*/*z* 171) are formed for both the protonated epoxyeudesmanolides** 1** and** 2**, their formations depend on the position of the epoxide ring. For compound** 1**, the oxygen lone pair is involved in a Grob-Wharton-type fragmentation, which results in the cleavage of the C(2)–C(3) bond and consequent formation of the intermediate ion** H4**. Further heterolytic cleavage of the C(4)–O produces a terminal carbonyl group (i.e., an aldehyde), from which the CO is eliminated to form the product ion** K1** (*m*/*z* 171). In contrast, formation of** K** for compound** 2** is initiated by the conversion of** H1** into the intermediate ion** H6** by means of a rearrangement assisted by the oxygen lone pair of the epoxide ring. Cleavage of the C(4)–O produces** H7**, from which the CO elimination occurs to produce** K2** (*m*/*z* 171) by a mechanism similar to that involved in the formation of** K1** from** H5** (Scheme 3).

### 3.4. Formation of the Diagnostic Ions D, G, I, and L

**Scheme 4:**
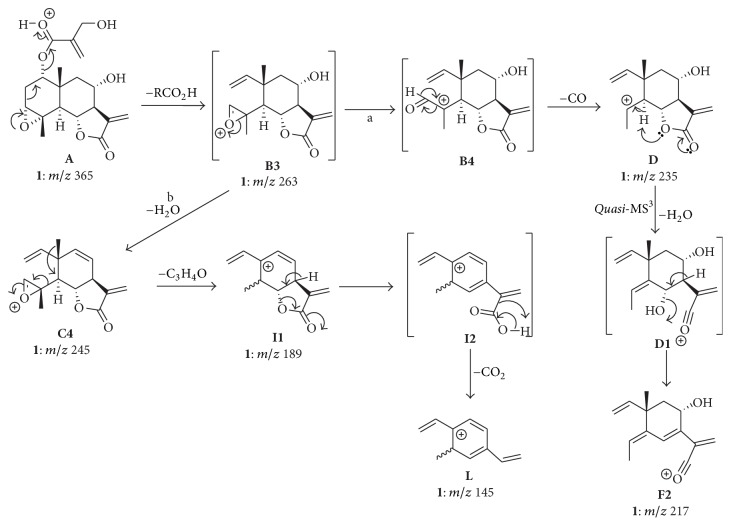
Formation of diagnostic product ions** D**,** I**, and** L **for compound** 1**.

The formation of the product ions** D** (*m*/*z* 235),** G** (*m*/*z* 209),** I** (*m*/*z* 189), and** L** (*m*/*z* 145), which are diagnostic for compound** 1**, is associated with the C(3)–O bond. This bond places the oxygen of the epoxide ring at a particular position of the eudesmanolide skeleton that propitiates the direct participation of the oxygen lone pair in the elimination of the ester side chain at C(1). By means of the Grob-Wharton fragmentation depicted in Scheme 4, the 3,4-epoxide ring can play a key role in the formation of** B** through another pathway compared to that proposed in Scheme 2. The heterolytic cleavage of the C(4)–O bond of the resulting product ion** B3** (*m*/*z* 263) produces the intermediate ion** B4** (pathway a, Scheme 4). Elimination of CO from the aldehyde moiety of** B4** produces** D** (*m*/*z* 235), which is diagnostic for the epoxide ring between C(3) and C(4). Further opening of the *γ*-lactone leads to the formation of the intermediate acylium ion** D1**, which decomposes into** F2** (*m*/*z* 217). In the case of compound** 1**, the product ion** F** can be formed through two different pathways, as shown in Schemes 2 and 4. This could explain, at least in principle, the higher relative intensity of this ion in the CID spectrum of** 1** as compared to** 2**. On the other hand, pathway b involves the elimination of H_2_O from** B3** and consequent formation of** C4** (*m*/*z* 245). The elimination of 2-methyloxirene (C_3_H_4_O) by means of the heterolytic cleavage of the C(4)–C(5) and further 1,2-methyl shift from C(10) to C(5), which is driven by the charge stabilization, caused** I1** (*m*/*z* 189). Further remote hydrogen rearrangement results in the opening of the *γ*-lactone ring and produces the intermediate ion** I2**, which has extended conjugation as compared to** I1**. Elimination of CO_2_ from** I2 **(44 u) results in the product ion** L **(*m*/*z* 145), which is also diagnostic for the epoxide ring between C(3) and C(4).

**Scheme 5:**
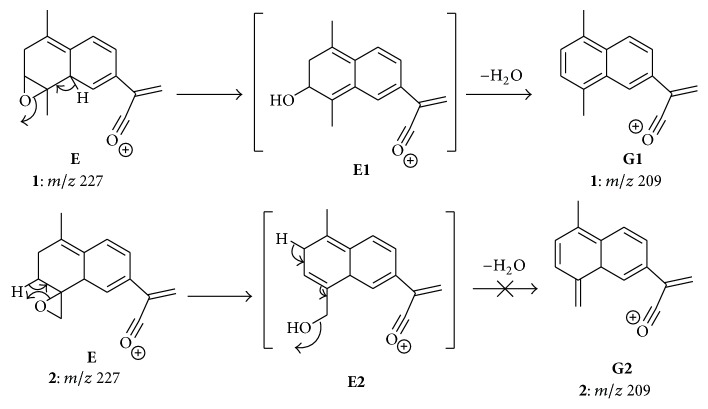
Formation of the diagnostic product ion** G **for compound** 1**.

Finally, the product ion** G** (*m*/*z* 209) from** E** can also be considered diagnostic for presence of epoxide ring at position 3,4. For compound** 1**, its formation involves opening of the epoxide ring by a remote hydrogen rearrangement and further elimination of H_2_O (Scheme 5). A similar pathway could also lead to the formation of** G** for compound** 2**. However, the aromaticity of** G1** makes it much more stable than** G2**, which displays an exocyclic double bond between C(4) and C (15). Therefore, the formation of** G1** is much more favored energetically than the formation of** G2**, so that the product ion of *m*/*z* 209 is observed only in the product ion spectrum of** 1.**

## 4. Conclusions

In summary, we have identified a series of diagnostic ions for compound** 1** that could be useful for the fast differentiation of 3,4- and 4,15-epoxyeudesmanolides** 1** and** 2 **by ESI-MS/MS, as an alternative to NMR. Moreover, these results could be also applied to the selective quantitation of these compounds directly from* Dimerostemma* extracts by liquid chromatography-tandem mass spectrometry (LC-MS/MS) using the transition of the precursor ion to these diagnostic ions in multiple reaction monitoring (MRM) methodologies. However, further studies with a wide range of epoxyeudesmanolides should be addressed to verify the potential of ESI-MS/MS to identify other epoxyeudesmanolides derivatives.

## Figures and Tables

**Table 1:** Assignments of the main product ions of the protonated epoxyeudesmanolides **1** and **2 ** and their respective relative intensities.

Assignment	**1**	**2**
**A** ([M + H]^+^)	365 (31)/368	379 (29)/381
**B** (**A** − R_1_OH)	263 (40)/264	263 (23)/264
**C** (**B **− H_2_O)	245 (100)/245	245 (100)/245
**D** (**B **− CO)	235 (12)/236^*∗*^	—
**E** (**C **− H_2_O)	227 (87)/227	227 (42)/227
**F** (**C **− CO and **D **− H_2_O)	217 (62)/218	217 (19)/218
**G (E **− H_2_O**)**	209 (<5)/^*∗*^	*∗*
**H** (**E **− CO and **F **− H_2_O)	199 (67)/199	199 (81)/199
**I (F **−** CO)**	189(5)/^*∗*^	189 (<5)/^*∗*^
**J** (**H **− H_2_O)	181 (56)/181	181 (21)/181
**K** (**H **− CO)	171 (21)/^*∗*^	171 (23)/^*∗*^
**L **(**I** − CO)	145 (10)/^*∗*^	*∗*

^*∗*^Relative intensity lower than 5%. The relative intensities are given between parentheses. Data from the experiments with labelled hydrogen ([M + 4D]^+^ for **1** and [M + 3D]^+^ for **2**) are given after bars.
